# Compliance and clinical efficacy of vaginal dilator after radiotherapy for cervical and endometrial malignancies

**DOI:** 10.3332/ecancer.2023.1545

**Published:** 2023-05-04

**Authors:** Rabia Tahseen, Yumna Ahmed, Maria Tariq, Sehrish Abrar, Nasir Ali

**Affiliations:** Section of Radiation Oncology, Department of Oncology, Aga Khan University, Karachi 74800, Pakistan

**Keywords:** vaginal dilator, pelvic radiation therapy, endometrial cancer, cervical cancer, compliance

## Abstract

**Objective:**

To investigate the compliance and clinical efficacy of vaginal dilators (VDs) as an educational intervention in patients receiving pelvic radiation therapy (RT) for endometrial and cervical malignancies.

**Material and methods:**

This is a single institution, retrospective chart review. Patients undergoing pelvic RT for endometrial or cervical cancer at our center were educated about the use of a VD starting 1 month after completion of RT. The patients were assessed after 3 months of prescribing VD. The demographic details and physical examination findings were extracted from medical records.

**Results:**

We identified 54 female patients at our institution during the 6-month duration. The median mean age of patients was 54 ± 9.9 years. Twenty-four (44.4%) had endometrial cancers and 30 (55.6%) were diagnosed with cervical cancers. All patients received external beam RT, 38 (70.4%) received a dose of 45 Gy, and 16 (29.6%) patients received 50.4 Gy. Brachytherapy was also received by all patients, 28 (51.9%) received 5 Gy × 2 fractions, 4 (7.4%) received 7 Gy × 3 fractions and 22 (40.7%) received 8 Gy × 3 fractions. The compliance with VD use was 36 (66.6%) patients. Twenty-two (40.7%) used 2–3 times a week, 8 (14.8%) used <2 times per week and 6 (11.9%) used only once a month, and 18 (33.3%) did not use the VD post-treatment. Per vaginal (PV) examination findings of the patient’s vagina with normal mucosa were evaluated in 32 (59.3%) and adhesions were found in 20 (37.0%) and 2 (3.7%) were unable to examine due to dense adhesions. During examination 12 (22.2%) had bleeding PV, however, the majority of the patients, 42 (77.8%) experienced no bleeding PV. Out of the 36 patients who used a VD, it was found to be efficacious in 29 (80.6%) of patients. Upon stratification of efficacy with a frequency of VD, 72.4% (*n* = 21) efficacy was seen in patients using frequent VD as prescribed 2–3 times per week.

**Conclusion:**

The compliance and efficacy of VD use after radiation to pelvic in cervical and endometrial cancers at 3 months follow-up were found to be 66.6% and 80.6%, respectively. This shows that VD therapy is an effective interventional tool and patients should receive specialist education about vaginal stenosis as toxicity at the outset of treatment.

## Introduction

Gynaecological malignancies are one of the most common malignancies in women, mainly comprising cervical and uterine cancer. The incidence has declined in developed countries, but it is on the rise in developing countries due to a lack of awareness, effective screening and treatment of pre-invasive lesions and low priority of women’s health [1]. The standard treatment of gynaecological malignancy is radiation therapy (RT) to the pelvis which can be external and/or internal (brachytherapy) and may be given alone or in combination with surgery and/or chemotherapy [2, 3]. The side-effect profile of pelvic RT includes acute dermatologic, genito-urinary, gastrointestinal and vaginal toxicity [4]. Radiation to the pelvis results in direct damage to the vaginal mucosa, connective tissues and small blood vessels with subsequent stenosis which leads to dryness, atrophy of the vagina, thinning of epithelium and development of fibrosis [5, 6].

These side effects lead to impairment in quality of life and sexual function. It also makes clinical per-vaginal (PV) examination difficult and intolerable. The importance of stenosis and pain-free examination is because most recurrences occur in the vagina, and difficult examination would hamper adequate clinical examination leading to missed detection of recurrences [7, 8].

Introducing any therapy which will minimise the impact of radiation damage might lead to sexual recovery for women after treatment. To prevent this toxicity, an educational intervention has been recommended by National Gynecological Oncological Nurse Forum and the American Cancer Society to use a vaginal dilator (VD) 2–3 times a week for an indefinite period to minimise the complications [9, 10].

The incidence of vaginal stenosis (VS) is reported as varying from 1.2% to 88% [11]. However, a low compliance rate has been reported in some studies that evaluated sequelae of pelvic RT [12, 13].

This study aims to determine the compliance and clinical efficacy of VDs in patients receiving pelvic RT for endometrial and cervical malignancies as there is a scarcity of local data in this regard.

## Methodology

This is a single institution, retrospective chart review to determine the compliance and clinical efficacy of VD. Institutional Ethical Review Committee’s approval was obtained. All patients with biopsy-proven cervical and endometrial cancer referred by a gynae-oncology surgeon or medical oncologist for radical radiotherapy were enrolled. A report of 6 months from 1st January to 30th June 2020 was documented. The inclusion criteria were a) patients receiving external beam RT (EBRT) and brachytherapy; b) less than 75 years and c) VD were prescribed by the radiation oncologist prescribed 1 month following RT. Patients were instructed by nursing staff about the use of VD three times a week, starting 4–6 weeks after delivery of last fraction of radiotherapy. Patients with previous history of radiotherapy in the same region, recurrent disease or metastatic disease were excluded from this study.

The following details were extracted from the medical records: age, site of disease, treatment received, and frequency of use of VD, bleeding PV during examination and PV exam findings at 3 months follow-up after prescribing the VD.

### VS and adhesions

As per the operational definition, VS and adhesions were evaluated on PV examination in the clinic on follow-up. The ability to pass two fingers and no adhesions were considered as a VD’s efficacy as this method was considered reproducible by Nunns *et al* [14]. On the contrary, the inability to pass two fingers in the vagina along with the formation of adhesions was considered as the ineffectiveness of the VD. Data were collected by the researcher from hospital medical records and documented as required in the study proforma.

### Data analysis

Statistical Package for the Social Sciences (version 23.0) was used for statistical analysis. A descriptive analysis was carried out. Mean and standard deviation were calculated for quantitative *variables*, i.e., age, EBRT dose and brachytherapy dose. Percentages were calculated for qualitative variables, i.e., diagnosis, surgery status, chemotherapy received, frequency of VD use, PV examination findings, bleeding after PV exam and efficacy of VD.

Stratification of outcome variables was performed for effect modifiers like frequency of VD and PV findings. Post-stratification chi-square test was applied. A *p*-value less than or equal to 0.05 was considered statistically significant.

## Results

**Figure 1.**
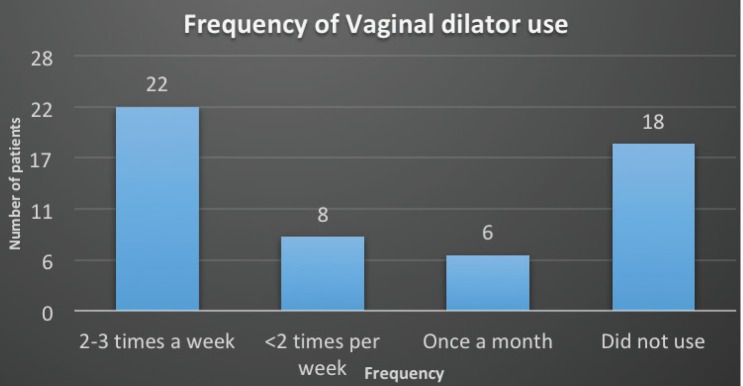
Frequency of VD use.

We identified 54 female patients at our institution during the 6 months of duration. Table 1 summarises the patient and treatment characteristics. The median mean age of patients was 54 ± 9.9 years. Twenty-four (44.4%) had endometrial cancers and 30 (55.6%) were diagnosed with cervical cancers. All patients received EBRT, 38 (70.4%) received 45 Gy and 16 (29.6%) patients received 50.4 Gy. Brachytherapy was also received by all patients 28 (51.9%) received 5 Gy × 2 fractions, 4 (7.4%) received 7 Gy × 3 fractions and 22 (40.7%) received 8 Gy × 3 fractions. The compliance with VD use was 36 (66.6%). Twenty-two (40.7%) used 2–3 times a week, 8 (14.8%) used <2 times per week and 6 (11.9%) used only once a month (Figure 1).

**Figure 2.**
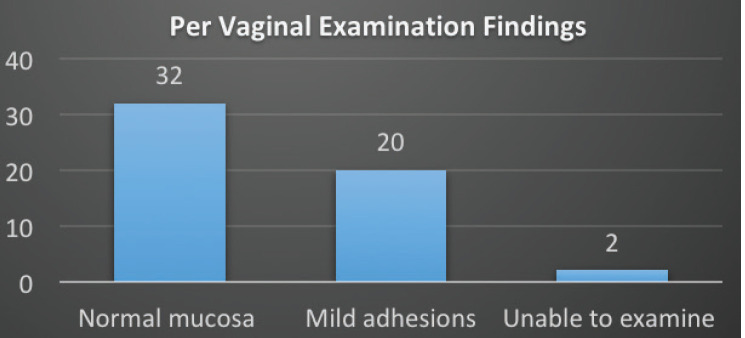
PV examination findings.

PV exam findings of the patient’s vagina and normal mucosa were seen in 32 (59.3%) and adhesions were found in 20 (37.0%) and 2 (3.7%) were unable to examine due to dense adhesions (Figure 2).

**Figure 3.**
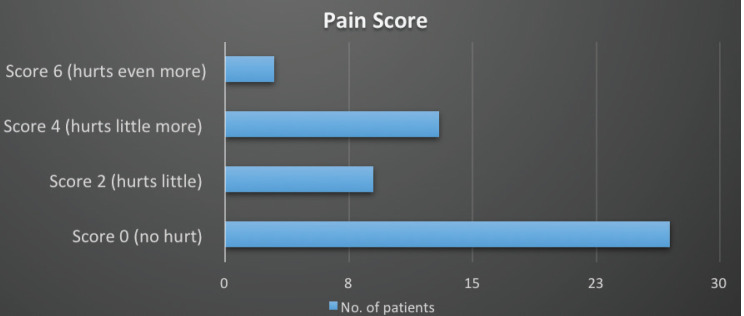
Pain score.

A pain score of 0 (no hurt) was seen in 27 (50.0%), a pain score of 2 (hurts little) in 9 (16.7%) pain score of 4 (hurts little more) in 13 (17.1%) and a pain score 6 ((hurts even more) in 3 (5.6%) patients (Figure 3).

During examination 12 (22.2%) had bleeding PV, however, the majority of patients 42 (77.8%) experienced no bleeding PV. Out of the 36 patients who used a VD, it was found to be efficacious in 29 (80.6%) of patients. Upon stratification of efficacy with the frequency of VDs, the efficacy of 72.4% (*n* = 21, *p*-value 0.03) was seen in patients using frequent VDs as prescribed 2–3 times per week (Table 2). Stratification of PV finding with the frequency of dilation revealed that patients who used VD for 2–3 times/week had normal mucosa on PV finding in 65.6% of patients.

**Figure 4.**
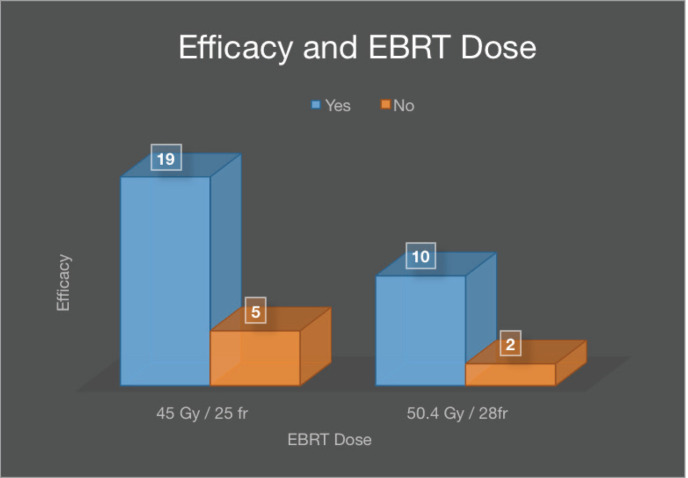
Efficacy with respect to EBRT dose.

Although the results are statistically insignificant, stratification of efficacy with age groups revealed 13.8% (4) efficacy in those under 40 years of age, 44.8% (13) in 41–60 years, and 41.4% (12) in 61 years and above (Table 3). Efficacy in patients receiving an EBRT dose of 45 Gy was 79.2% (19) and 34.5% (10) in 50.4 Gy as shown in Figure 4.

**Figure 5.**
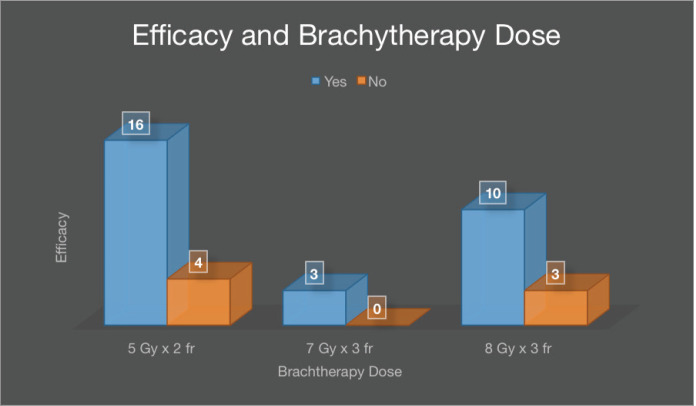
Efficacy with respect to brachytherapy dose.

Figure 5 shows efficacy in patients receiving a brachytherapy dose of 5 Gy × 2 fractions was 55.2% (16), 7 Gy × 3 fractions 10.3% (3) and 8 Gy × 3 fractions arm 34.5% (10) which is not statistically insignificant.

## Discussion

VS is a well-known side effect of pelvic RT. The reported incidence of VS resulting from RT varies, with an overall range of 2.5%–88% [15, 16]. Most of the evidence used to support the current estimates of the prevalence of VS comes from retrospective studies with small cohorts, and there are significant differences across researchers in terms of measurement methods and reported grading of VS [17].

The clinical impact of VS is intricate and multifaceted. If VS develops, surveillance of the vagina and cervix and pelvic examination can be limited by post-treatment scarring and may be painful or uncomfortable for the patient. Since the pattern of failure of cervical and endometrial cancer is vaginal vault and mucosa, the patency of the vagina during examination is pertinent [18]. Psychologically, pain with sexual intercourse or PV examinations may trigger distressing or even traumatic memories for a patient of her cancer and treatments. The VDs are smooth cylindrical devices that come in a set of four different sizes and are made of plastic or silicon, should be used as directed by the physician to prevent the shortening and narrowing of the vagina. Observational data, however, show that routine dilator use after RT is linked to decreased incidence of VS [19]. The aim of VD therapy (VDT) in the early phase after pelvic RT is to prevent the formation of adhesions between the vaginal mucosa walls which may later form dense, thick adhesions nonamenable to adhesiolysis and may prevent proper PV examination. The other important purpose of the dilator is to antagonise the late effects after RT in the submucosa including circumferential fibrosis and elastosis of the vagina by stretching the mucosal walls and promoting epithelial growth. A few observational studies have found significant associations between dilator use and a lower risk of VS after RT. Decruze *et al* [20], reported VS of 11% versus 57% of patients who did use versus did not use a custom-made vaginal stent had VS at follow-up. In contrast, two randomised studies in which despite vaginal dilation, no improvement was observed in sexual function scores of women [21, 22].

Despite varied evidence, VDT is a pertinent tool along with hormonal therapies, moisturisers and lubricants in maintaining vaginal and sexual function after treatment of cancer. Guidelines have proposed strategies for standardising and promoting VDT education, methods and techniques.

Many radiation oncologists acknowledge this as their responsibility in managing the vaginal and sexual health of patients after pelvic RT. But still may need further practical training and education. The lack of standards in terms of how the optimal duration of dilator use, and monitoring of such intervention may be a crucial component to determining what is effective, acceptable or feasible for a patient on an individual basis. The frequency and duration of dilator use, the ideal time interval after pelvic RT in which to begin the usage, the size of the dilator and insertion technique, and the necessity of dilators in sexually active patients are all areas where there is a lack of agreement [23]. Unsatisfactory dilator compliance is a significant problem that physicians deal with. Despite prescribing the dilator patients chose not to use it because it might be uncomfortable for them. Compliance with VDs has been reported as poor in two studies, with only 14%–26% using the dilator as directed at 6 months as directed and only 6%–12% using it as directed at 12 months [24, 25].

In our study, compliance with dilators at 3 months was 66.6% and another study by Brand and Stenlake [13] reported at 12 months, 52% of patients were still using the dilators, and 35% were using the dilators at least 2–3 times per week.

The efficacy discussed in our study is dependent upon a patient’s vagina with normal mucosa and pain-free examination, which was seen as consistent with patients using dilators regularly. Although our study may provide some low-level evidence of the benefit of dilators in maintaining vaginal health, efficacy, and preventing stenosis, there is clearly a need for a larger study comprising randomised controlled trials to address this pertinent side effect of RT which hinders in patients’ well-being psychologically and physiologically.

The first systematic review evaluating compliance to VDs reported a low compliance rate due to psychological issues and inability to use the dilator which is consistent with our study in which the dropout rate was 33% [23]. However, this was the limitation of our study in which reasons for low compliance were not evaluated.

According to some data, the usage of VDs may be hampered by the lack of an objective VS measurement [23]. Therefore, further in-depth longitudinal research is needed to address this problem. The generalisability of these conclusions is constrained by the dearth of research and their heterogeneity. It is important to take into account the clinical and methodological variability that may cause variations in treatment results. Women may perceive dilators and their adverse effects differently, which may lead to variations in compliance. Women’s reluctance to use dilators may be an attempt at avoiding unpleasant emotions like mental or physical discomfort or to alleviate humiliation and shame, preventing further harm to their sense of self.

The limitations of the study are the lack of a control group in our retrospective analysis which makes it challenging for us to determine whether it was our educational intervention of dilator therapy that led to improved efficacy and reduced stenosis. Long-duration follow-up was not done due to the limitation of time and despite our exhaustive search of local studies on the use of VDs and their efficacy and compliance from lower middle-income countries, which could not be incorporated into our study.

## Conclusion

The compliance and clinical efficacy of VD use after radiation to pelvic in cervical and endometrial cancers at 3 months follow-up were found to be 66.6% and 80.6%, respectively. This demonstrates the value of VDT as an effective interventional tool and highlights the need for patients to get specialised education about VS at the outset of treatment. More significantly, guidance about VS and routine evaluations of vaginal toxicity ought to be given during the post-treatment surveillance period.

## Conflicts of interest

The author(s) declare that they have no conflict of interest.

## Funding

No funds, grants, or other support were received.

## Figures and Tables

**Table 1.** Patient and treatment characteristics.

Characteristics	*n* (%)
Patients	54
Age (years)	Mean 54 ± 9.9
Age groups: (years) ≤ou 41–60 61+	6 (11.1%) 30 (55.6%) 18 (33.3%)
Anatomic site of disease Endometrium Cervix	24 (44.4%) 30 (55.6%)
Surgery Yes No	31 (57.4%) 23 (42.6%)
Chemotherapy Yes No	37 (68.5%) 17 (31.5%)
EBRT dose 45 Gy in 25 fractions 50.4 Gy in 28 fractions	38 (70.4%) 16 (29.6%)
Brachytherapy dose 5 Gy × 2 fractions (10 Gy) 7 Gy × 3 fractions (21 Gy) 8 Gy × 3 fractions (24 Gy)	28 (51.9%) 4 (7.4%) 22 (40.7%)

**Table 2.** Stratification of efficacy in compliant patients with frequency of dilator.

Efficacy		Frequency of dilator				*p* value
		2–3 times/ week	<2 times/ week	Once a month	Total	
Yes	Count % within efficacy % within frequency of dilator	21 72.4% 95.5%	6 20.7% 75.0%	2 6.9% 33.3%	29 100% 80.6%	0.03
No	Count % within efficacy % within frequency of dilator	1 14.3% 4.5%	2 28.6% 25.0%	4 57.1% 66.7%	7 100% 19.4%	

**Table 3.** Stratification of efficacy in compliant patients with age groups.

Efficacy	Age groups % (*n*)			*p* value
	<40 years	41–60 years	61+ years	
Yes	13.8% (4)	44.8% (13)	41.4% (12)	NS
No	14.3% (1)	57.1% (4)	28.6% (2)	

## References

[1] Ansink AC (2007). Cervical cancer in developing countries: how can we reduce the burden? Awareness raising, screening, treatment and palliation. Trop Doct.

[2] de Boer SM, Powell ME, Mileshkin L (2018). Adjuvant chemoradiotherapy versus radiotherapy alone for women with high-risk endometrial cancer (PORTEC-3): final results of an international, open-label, multicentre, randomised, phase 3 trial. Lancet Oncol.

[3] Greven K, Winter K, Underhill K (2006). Final analysis of RTOG 9708: adjuvant postoperative irradiation combined with cisplatin/paclitaxel chemotherapy following surgery for patients with high-risk endometrial cancer. Gynecol Oncol.

[4] Hafiz A, Abbasi AN, Ali N (2015). Frequency and severity of acute toxicity of pelvic radiotherapy for gynecological cancer. J Coll Physicians Surg Pak.

[5] Yoshida K, Yamazaki H, Nakamura S (2015). Role of vaginal pallor reaction in predicting late vaginal stenosis after high-dose-rate brachytherapy in treatment-naive patients with cervical cancer. J Gynecol Oncol.

[6] Varytė G, Bartkevičienė D (2021). Pelvic radiation therapy induced vaginal stenosis: a review of current modalities and recent treatment advances. Medicina.

[7] Creutzberg CL, Nout RA, Lybeert ML (2011). Fifteen-year radiotherapy outcomes of the randomized PORTEC-1 trial for endometrial carcinoma. Int J Radiat Oncol Biol Phys.

[8] Huh WK, Straughn JM, Mariani A (2007). Salvage of isolated vaginal recurrences in women with surgical stage I endometrial cancer: a multiinstitutional experience. Int J Gynecol Cancer.

[9] Hanlon A, Small Jr W, Strauss J (2018). Dilator use following vaginal brachytherapy for endometrial cancer: a randomized feasibility and adherence study. Cancer Nurs.

[10] Stahl JM, Qian JM, Tien CJ (2019). Extended duration of dilator use beyond 1 year may reduce vaginal stenosis after intravaginal high-dose-rate brachytherapy. Support Care Cancer.

[11] Brand AH, Bull CA, Cakir B (2006). Vaginal stenosis in patients treated with radiotherapy for carcinoma of the cervix. Int J Gynecol Cancer.

[12] Akbaba S, Oelmann-Avendano JT, Krug D (2019). The impact of vaginal dilator use on vaginal stenosis and sexual quality of life in women treated with adjuvant radiotherapy for endometrial cancer. Strahlenther Onkol.

[13] Brand AH, Do V, Stenlake A (2012). Can an educational intervention improve compliance with vaginal dilator use in patients treated with radiation for a gynecological malignancy?. Int J Gynecol Cancer.

[14] Nunns D, Williamson K, Swaney L The morbidity of surgery and adjuvant radiotherapy in the management of endometrial carcinoma. Int J Gynecol Cancer.

[15] Thakur P, Dogra E, Gupta M (2019). Comparison of iso-effective and cost-effective high-dose-rate brachytherapy treatment schedules in cervical cancer–regional cancer center experience. J Contemp Brachyther.

[16] Hartman P, Diddle AW (1972). Vaginal stenosis following irradiation therapy for carcinoma of the cervix uteri. Cancer.

[17] Mirabeau-Beale K, Hong TS, Niemierko A (2015). Clinical and treatment factors associated with vaginal stenosis after definitive chemoradiation for anal canal cancer. Pract Radiat Oncol.

[18] Gadducci A, Cosio S, Fabrini MG (2011). Patterns of failures in endometrial cancer: clinicopathological variables predictive of the risk of local, distant and retroperitoneal failure. Anticancer Res.

[19] Law E, Kelvin JF, Thom B (2015). Prospective study of vaginal dilator use adherence and efficacy following radiotherapy. Radiother Oncol.

[20] Decruze SB, Guthrie D, Magnani R (1999). Prevention of vaginal stenosis in patients following vaginal brachytherapy. Clin Oncol.

[21] Jeffries SA, Robinson JW, Craighead PS (2006). An effective group psychoeducational intervention for improving compliance with vaginal dilation: a randomized controlled trial. Int J Radiat Oncol Biol Phys.

[22] Robinson JW, Faris PD, Scott CB (1999). Psychoeducational group increases vaginal dilation for younger women and reduces sexual fears for women of all ages with gynecological carcinoma treated with radiotherapy. Int J Radiat Oncol Biol Phys.

[23] Haddad NC, Soares Brollo LC, Pinho Oliveira MA (2021). Diagnostic methods for vaginal stenosis and compliance to vaginal dilator use: a systematic review. J Sex Med.

[24] Schover LR, Fife M, Gershenson DM (1989). Sexual dysfunction and treatment for early stage cervical cancer. Cancer.

[25] Robinson JW, Scott CB, Fans PD (1994). Sexual rehabilitation for women with gynecological cancer: information is not sufficient. Can J Hum Sex.

